# The healthy eating index may not be an appropriate indicator for assessing dietary quality in breast cancer survivors: results from NHANES 2005–2018

**DOI:** 10.3389/fnut.2024.1519607

**Published:** 2024-12-30

**Authors:** Hao Zheng, Siyang Chen, Lihua Huang, Xiao Zhou, Qingxi Huang, Xuemei Li, Yanli Zhao

**Affiliations:** State Key Laboratory of Oncology in South China, Guangdong Key Laboratory of Nasopharyngeal Carcinoma Diagnosis and Therapy, Guangdong Provincial Clinical Research Center for Cancer, Sun Yat-sen University Cancer Center, Guangzhou, China

**Keywords:** breast cancer, cancer survivors, dietary quality, healthy eating index, mortality, NHANES

## Abstract

**Background:**

Evidence on the relationship between the Healthy Eating Index (HEI) and mortality in breast cancer (BC) survivors remains inconclusive. Moreover, rare studies have explored the effect of individual HEI components on survival in this population. This study explored the association between the HEI-2020, including total and 13 component scores, and mortality in BC survivors.

**Methods:**

This cross-sectional study included data of 481 female BC survivors (representing a 3.3 million population) obtained from the National Health and Nutrition Examination Survey (NHANES) 2005–2018. The HEI-2020 total and component scores (higher scores indicating superior dietary quality) were calculated based on the 24 h dietary recall interview. Data on mortality until December 31, 2019, were obtained from the NHANES Public-Use Linked Mortality File. The weighted Cox proportional hazards models were used to assess the association between HEI-2020 and mortality outcomes.

**Results:**

After fully adjusting for confounders, a qualified total HEI-2020 score (≥60) was significantly associated with reduced non-cancer mortality (HR 0.59, 95%CI: 0.35–0.99), but not with all-cause or cancer-specific mortality. Among the 13 HEI components, a lower intake of added sugars (with a qualified component score) was linked to a decreased risk of both all-cause and non-cancer mortality (HR 0.44 and 0.25, 95%CI: 0.25–0.77 and 0.13–0.48, respectively, all *p* < 0.05). Conversely, higher consumption of seafood and plant proteins (with a qualified component score) correlated with an increased risk of cancer-specific mortality (HR 3.64, 95%CI: 1.57–8.45), and a higher intake of dairy was associated with an elevated risk of both all-cause and non-cancer mortality (HR 2.12 and 2.81, 95%CI: 1.36–3.29 and 1.56–5.07, respectively).

**Conclusion:**

Higher total and component scores of the HEI-2020 do not uniformly confer a lower mortality risk for BC survivors. The HEI-2020 may not be an appropriate indicator for post-diagnosis dietary assessment or recommendations for BC survivors.

## 1 Introduction

Breast cancer (BC) is the most commonly diagnosed cancer among women and continues to pose a significant public health challenge worldwide. In 2022, there were approximately 2.3 million new cases of female BC, accounting for 11.6% of all newly diagnosed cancer cases globally (1). Over recent decades, advancements in early detection and treatment modalities have contributed to a steady increase in long-term survival rates for BC patients, with recent statistics indicating that 91.2% of BC patients survive for 5 years or longer (2, 3). This improvement in survival rates has led to a notable increase in the population of BC survivors worldwide, who frequently encounter distinct challenges in maintaining their health and mitigating the risks of recurrence and mortality following diagnosis (4). As of January 1, 2022, there were approximately 4.1 million BC survivors in the United States (5), presenting a significant challenge for healthcare providers and policymakers.

A substantial body of research has identified positive associations between high-quality diets and improved mortality outcomes among cancer survivors, suggesting that dietary interventions may enhance survival by influencing insulin and glucose metabolism, bolstering immune function, regulating hormone metabolism, reducing inflammation, and inhibiting tumor growth and metastasis (6–9).

Although data reveal that some BC survivors have modified their dietary behaviors after BC diagnosis—such as increasing their consumption of fruits and vegetables, while decreasing fat intake (10, 11), studies showed the dietary quality (DQ) of BC survivors remains suboptimal (12). This may be attributed to the absence of dietary guidelines specifically tailored for BC survivors and the lack of validated metrics to evaluate DQ within this demographic.

In the United States, DQ is evaluated based on adherence to the Dietary Guidelines for Americans (DGA). The HEI serves as a scoring metric for overall DQ as well as the quality of several dietary components, independent of quantity, and can be used to assess alignment with the DGA (13). HEI has gained widespread application in surveillance, epidemiological, and intervention studies to analyze DQ within populations, explore the associations between DQ and health outcomes, and evaluate the effects of interventions on DQ, as well as in economic and food environment-related research (14). The HEI-2020 consists of 13 components that embody a healthy eating pattern, including fruits, vegetables, grains, dairy, proteins, and more. Each component is assigned a score based on intake relative to the criteria established by the DGA, thereby providing a quantitative assessment of DQ (13).

Although the HEI is widely used for DQ assessment in healthy populations, there exists limited and inconsistent evidence regarding the relationship between the HEI and mortality outcomes among BC survivors. George et al. reported significant associations between higher HEI-2005 scores and lower all-cause and cancer-specific mortality (HR 0.40 and 0.12) among 670 BC survivors over a 6-year follow-up in 2011. However, they did not observe similar significant results in cancer-specific mortality among 2,317 postmenopausal BC survivors in 2014, while a significant association between higher HEI-2005 scores and non-cancer mortality (HR 0.58) was found (15, 16). Additionally, a study based on NHANES III found a significant correlation between a qualified HEI score and reduced all-cause mortality (HR 0.49, 95%CI 0.25–0.97) (17), and another study in 2018 found that lower HEI scores were associated with increased cancer-specific mortality (HR 1.66, 95% CI 1.09–2.52) (18). Conversely, Ergas et al. identified only an inverse association between HEI scores and non-cancer mortality, with no significant findings related to all-cause or cancer-specific mortality (19). It is important to note that prior studies have primarily focused on the relationship between the total HEI score and mortality outcomes, with limited studies delving into individual HEI components and their associations with mortality (15). Given the limited and inconsistent findings, the applicability of the HEI for DQ assessment in BC survivors remains uncertain.

As the population of BC survivors continues to grow, there is an increasing necessity to formulate more specific dietary recommendations that cater to the unique needs of this group. The current study aims to examine the association between DQ as assessed by the HEI-2020, including total and 13 components scores, and mortality in BC survivors to inform clinical practice and public health policy.

## 2 Materials and methods

### 2.1 Population

This cross-sectional study utilized data from the National Health and Nutrition Examination Survey (NHANES), which encompasses the years 2005 to 2018. NHANES is a nationally representative, continuous survey targeting the non-institutionalized, civilian population of the United States, conducted by the National Center for Health Statistics (NCHS) under the auspices of the United States Centers for Disease Control and Prevention. The survey employs a complex, stratified, multistage probability cluster sampling design and disseminates publicly accessible survey data as two-year datasets. Comprehensive details of NHANES data collection and methodological procedures are described elsewhere (20). All participants or their proxies provided written informed consents, and the NHANES protocol, along with the publicly released de-identified data, received approval from the Research Ethics Review Board at NCHS.

**Figure 1:**
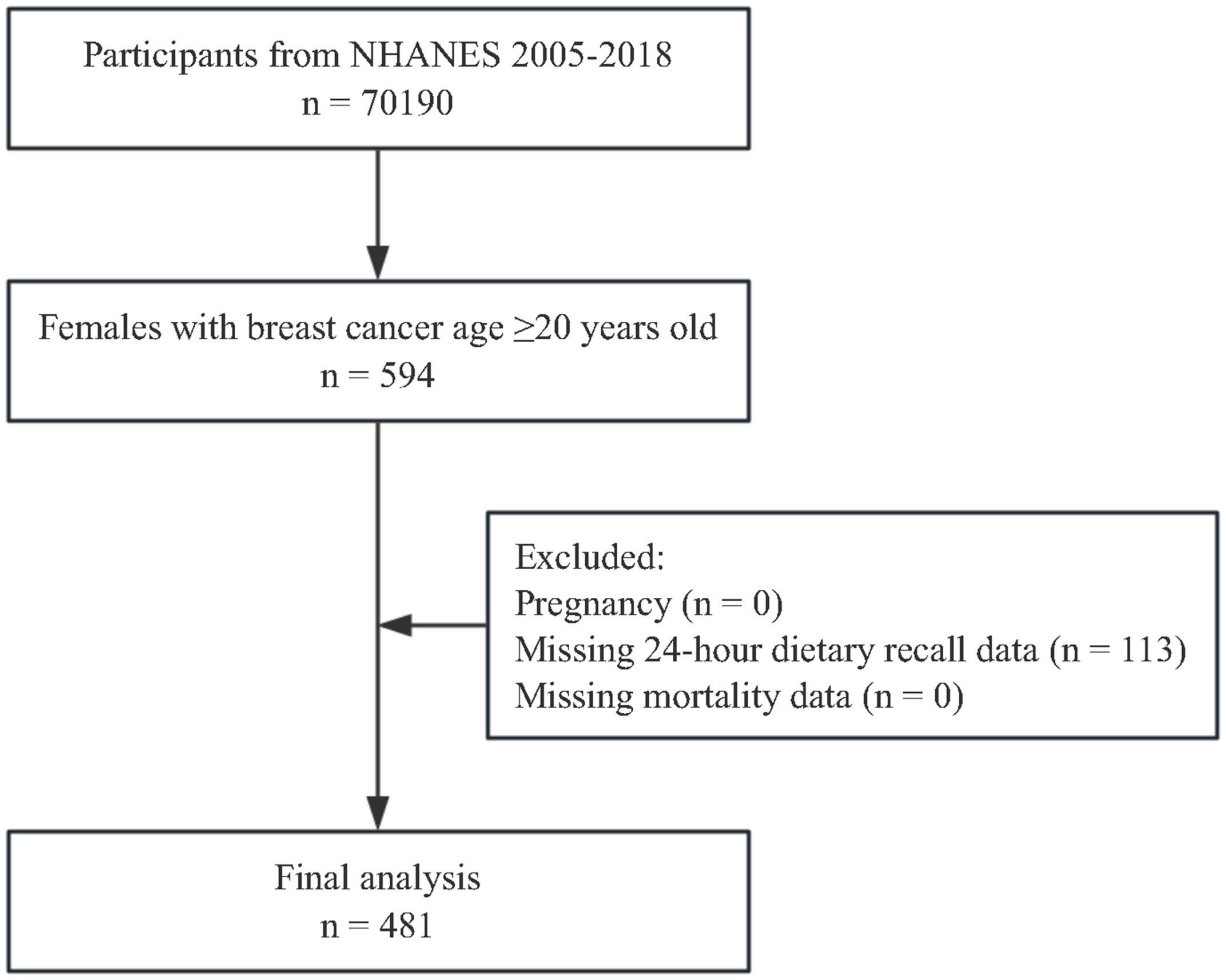
Flowchart of the study participants.

The present study focused on female adults aged 20 years and older who self-reported a prior diagnosis of BC. Of the 594 females with a history of BC, 113 were excluded from the analysis due to incomplete 24 h dietary recall data. As a result, the final unweighted sample included 481 BC survivors, representing a weighted population of 3,327,288. The participant selection process is outlined in Figure 1.

### 2.2 Dietary assessment

Post-diagnosis dietary information was collected through two 24 h dietary recall interviews. The data collection was strategically scheduled to encompass interviews conducted on all days of the week and throughout the year. During these interviews, trained interviewers prompted participants to report their food and beverage consumption over the past 24 h. Additional information including (but not limited to) the quantities, recipes, and dining locations was also documented. Nutrient values were derived from the ingredient nutrient values available in the United States Department of Agriculture’s (USDA) Survey Nutrient Database Nutrient Files. The dietary information obtained from the interviews was subsequently utilized to calculate the total and component scores of the HEI-2020 (21).

### 2.3 Healthy eating index-2020

The HEI-2020, developed by the USDA, serves as a scoring system to evaluate the overall quality of dietary intake. It was specifically designed to assess the extent to which the dietary habits of the U.S. population align with the 2020–2025 DGA. The HEI-2020 employs a density-based methodology and consists of 13 dietary components, which are categorized into 9 adequacy components (including total fruits, whole fruits, total vegetables, greens and beans, whole grains, dairy, total protein foods, seafood and plant protein, and fatty acids) and 4 moderation components (including refined grains, sodium, added sugars, and saturated fats). Generally, higher total HEI-2020 scores are indicative of superior DQ (range: 0–100). For the adequacy components (i.e., food components to encourage), elevated scores signify higher intake levels, while for the moderation components (i.e., food components to limit), higher scores indicate lower intake levels [22, 23]. Participants achieving a total HEI score of 60 are deemed to have adhered to the dietary guidelines (24). Consequently, we classified the total HEI-2020 scores into two classifications: unqualified (<60) and qualified (≥60). Similarly, for HEI components, a score is considered qualified when it meets or exceeds 60% of the maximum score for that component.

### 2.4 Outcome ascertainment

The outcome measures for this study encompass all-cause mortality, cancer-specific mortality, and non-cancer mortality. Data regarding mortality and follow-up duration were sourced from the NHANES Public-Use Linked Mortality File, published by the NCHS. This file links NHANES data with death certificate records from the National Death Index (NDI), ensuring that outcome data for the vast majority of participants are available and accurate, thereby significantly minimizing potential biases due to loss to follow-up and information errors. A comprehensive description of the linkage methodology of the file has been previously described (25). Prior research has confirmed the reliability of mortality ascertainment through the file (26–28). The follow-up time (measured in months) was defined as the interval from the completion of the NHANES questionnaire until the occurrence of death from any cause or the conclusion of the follow-up period (December 31, 2019), whichever event transpired first.

### 2.5 Covariates assessment

Participants provided information regarding their age, age at BC diagnosis, race (categorized as non-Hispanic White, non-Hispanic Black, Asian American, Mexican American, or other), educational level (less than high school, high school, or greater than high school), marital status (married/living with a partner, widowed/divorced/separated, or never married), family poverty income ratio (classified as <1.3, 1.3 to <3.5, or ≥ 3.5), smoking status (never, ever, or now), alcohol consumption (never, ever, or now), and moderate or vigorous physical activity (MVPA, engagement in any vigorous or moderate activity within the past week). The time interval between diagnosis and study entry was calculated as the difference between the age at the time of the survey and their age at BC diagnosis. The body-mass index (BMI) was computed as weight in kilograms divided by the square of height in meters, and participants were categorized into three weight-status groups: normal (BMI 18.5 to <25), underweight (BMI <18.5), overweight (BMI 25 to <30), or obese (BMI ≥ 30).

Diabetes diagnosis was established through self-reported history of diabetes or a hemoglobin A1c level of ≥6.5%. Hypertension was identified based on a systolic blood pressure of ≥130 mmHg, a diastolic blood pressure of ≥80 mmHg, or current use of antihypertensive medication. Participants were classified as having hyperlipidemia if they met any of the following criteria: total cholesterol ≥200 mg/dL, triglycerides ≥150 mg/dL, low-density lipoprotein cholesterol ≥130 mg/dL, or high-density lipoprotein cholesterol ≤50 mg/dL (29). Additionally, individuals taking lipid-lowering medications were also classified as having hyperlipidemia.

### 2.6 Statistical analysis

Sample weights were utilized to adjust for the complex survey design, thereby ensuring the generation of nationally representative estimates following the NHANES analytic guidelines. Baseline characteristics were presented based on the total HEI-2020 score classifications (unqualified and qualified). Continuous variables were reported as weighted means with standard errors (SE), while categorical variables were presented as unweighted sample counts with weighted percentages. Continuous variables exhibiting a normal distribution were analyzed using the student’s *t*-test, whereas those with a non-normal distribution were assessed using the Kruskal–Wallis test. Categorical variables were compared by using the chi-squared (χ^2^) test.

The relationship between HEI scores and mortality was evaluated through weighted Cox proportional hazards regression models. Model 1 represented the unadjusted analysis, while Model 2 included adjustments for age and race. Model 3 was the fully adjusted model, incorporated additional covariates, including age, race, marital status, educational level, family poverty income ratio, smoking status, alcohol use, BMI, hypertension, hyperlipidemia, diabetes, MVPA, and the time elapsed between diagnosis and study entry. The multiple imputation methods were used to handle the missing data (8.52% of family poverty income ratio, 3.12% of alcohol use, 2.08% of weight status, 0.62% of hyperlipidemia, 0.21% of smoking status) and sensitivity analyses were performed on participants with complete data only (*N* = 419). All statistical analyses were performed using R software (version 4.4.1), with a two-tailed *p*-value of <0.05 considered statistically significant.

## 3 Results

### 3.1 Study population characteristics

The study comprised a total of 481 BC survivors, representing a weighted population of 3,327,288, with a weighted median age of 65.4 years. Among the participants, 82.6% were identified as Non-Hispanic White, 59.8% reported never smoked, and only 9.5% were still smoking. Participants who achieved qualified HEI-2020 scores exhibited a longer time duration between diagnosis and study entry (13.8 years vs. 9.5 years), an extended follow-up time (89.5 months vs. 74.0 months), and a higher likelihood of engaging in MVPA within the past week (61.6% vs. 42.2%), compared with participants with unqualified scores. No statistically significant differences were observed in terms of age, age at diagnosis, educational level, marital status, income level, BMI categories, alcohol use, presence of comorbidities, or mortality categories between participants with qualified and unqualified total HEI scores (Table 1).

**Table 1:** Baseline characteristics of participants (*n* = 481).

Variable	Overall (*n* = 481)	Total HEI-2020 score		*p*-value
	*N* = 3,327,288^†^	Unqualified (*n* = 308)	Qualified (*n* = 173)	
		*N* = 2,144,518^†^	*N* = 1,182,770^†^	
Age, Mean (SE)	65.4 (0.9)	64.5 (1.2)	66.9 (1.2)	0.166
Age at diagnosis, Mean (SE)	54.3 (1.2)	55.0 (1.2)	53.1 (1.9)	0.364
Time between diagnosis and study entry, years, Mean (SE)	11.0 (0.8)	9.5 (0.7)	13.8 (1.3)	**0.001**
Follow-up time, months, Mean (SE)	79.5 (3.1)	74.0 (4.1)	89.5 (5.7)	**0.044**
Race, *n* (%)				**0.018**
Hispanic	78 (5.9)	53 (5.5)	25 (6.7)	
Non-Hispanic White	287 (82.6)	178 (82.6)	109 (82.6)	
Non-Hispanic Black	87 (8.2)	65 (10.1)	22 (4.8)	
Other Race	29 (3.3)	12 (1.8)	17 (5.9)	
Educational level, *n* (%)				0.652
Less than high school	100 (14.1)	69 (12.9)	31 (16.1)	
High school	110 (22.7)	76 (24.0)	34 (20.2)	
Greater than high school	271 (63.3)	163 (63.0)	108 (63.7)	
Marital status, *n* (%)				0.343
Married/Living with partner	243 (59.2)	148 (56.4)	95 (64.2)	
Widowed/divorced/separated	218 (38.0)	145 (40.9)	73 (32.8)	
Never married	20 (2.8)	15 (2.7)	5 (3.0)	
Family poverty income ratio, *n* (%)				0.565
<1.3	118 (14.8)	84 (14.6)	34 (15.2)	
1.3–3.49	194 (37.8)	129 (40.0)	65 (34.0)	
> = 3.5	169 (47.3)	95 (45.4)	74 (50.8)	
Weight status, *n* (%)				0.088
Normal	138 (28.9)	80 (27.1)	58 (32.2)	
Underweight	6 (1.1)	3 (0.9)	3 (1.5)	
Overweight	140 (30.6)	86 (27.0)	54 (37.3)	
Obese	197 (39.4)	139 (45.1)	58 (29.1)	
Smoking status, *n* (%)				**0.015**
Never	291 (59.8)	181 (60.8)	110 (58.0)	
Ever	146 (30.7)	88 (26.2)	58 (38.8)	
Now	44 (9.5)	39 (12.9)	5 (3.2)	
Alcohol use, *n* (%)				0.892
Never	107 (17.4)	67 (17.9)	40 (16.6)	
Ever	116 (19.7)	80 (20.2)	36 (18.8)	
Now	258 (62.9)	161 (61.9)	97 (64.6)	
MVPA, *n* (%)	202 (49.1)	110 (42.2)	92 (61.6)	**0.005**
Diabetes, *n* (%)	123 (22.2)	79 (20.4)	44 (25.4)	0.373
Hypertension, *n* (%)	342 (66.5)	226 (65.9)	116 (67.4)	0.810
Hyperlipidemia, *n* (%)	380 (80.2)	253 (81.4)	127 (77.8)	0.486
Mortality Status, *n* (%)				0.392
Alive	361 (80.7)	236 (81.9)	125 (78.6)	
Deceased	120 (19.3)	72 (18.1)	48 (21.4)	
Cancer mortality, *n* (%)	42 (6.7)	21 (5.4)	21 (9.1)	0.123
Noncancer mortality, *n* (%)	78 (12.5)	51 (12.7)	27 (12.3)	0.916

HEI-2020, Health Eating Index-2020; MVPA, moderate or vigorous physical activity. Data were presented as weighted mean (standard errors) or unweighted number (weighted percentages).^†^Weighted population. The bold *p*-values are less than 0.05, indicating statistical significance.

### 3.2 Association between HEI-2020 and all-cause mortality

After fully adjusted for confounders (model 3, Table 2), participants with qualified total HEI-2020 scores tend to have a lower risk of all-cause mortality compared to those with unqualified scores, but not statistically significant (HR 0.82, 95%CI: 0.56–1.21, *p* = 0.321).

**Table 2:** Association between HEI-2020 and all-cause mortality among breast cancer survivors (*n* = 481).

	Model 1		Model 2		Model 3	
	HR (95%CI)	*p-*value	HR (95%CI)	*p-*value	HR (95%CI)	*p-*value
Total HEI-2020 score						
Unqualified (<60)	Ref		Ref		Ref	
Qualified (≥60)	0.97 (0.64–1.46)	0.871	0.79 (0.56–1.12)	0.187	0.82 (0.56–1.21)	0.321
Adequacy components						
Total fruits						
Unqualified (<3)	Ref		Ref		Ref	
Qualified (≥3)	1.52 (1.00–2.32)	0.051	0.96 (0.65–1.43)	0.850	1.01 (0.68–1.50)	0.975
Whole fruits						
Unqualified (<3)	Ref		Ref		Ref	
Qualified (≥3)	1.11 (0.71–1.73)	0.639	0.82 (0.56–1.22)	0.335	0.90 (0.60–1.34)	0.592
Total vegetables						
Unqualified (<3)	Ref		Ref		Ref	
Qualified (≥3)	0.83 (0.47–1.44)	0.498	0.76 (0.49–1.19)	0.231	0.73 (0.42–1.25)	0.249
Greens and beans						
Unqualified (<3)	Ref		Ref		Ref	
Qualified (≥3)	0.72 (0.35–1.50)	0.387	0.79 (0.41–1.52)	0.485	0.87 (0.42–1.83)	0.718
Total protein foods						
Unqualified (<3)	Ref		Ref		Ref	
Qualified (≥3)	0.71 (0.33–1.55)	0.393	0.62 (0.33–1.18)	0.144	0.76 (0.37–1.56)	0.454
Seafood and plant proteins						
Unqualified (<3)	Ref		Ref		Ref	
Qualified (≥3)	0.99 (0.58–1.70)	0.974	1.10 (0.71–1.71)	0.667	1.38 (0.89–2.13)	0.150
Whole grains						
Unqualified (<6)	Ref		Ref		Ref	
Qualified (≥6)	0.98 (0.56–1.69)	0.932	0.75 (0.46–1.20)	0.231	0.67 (0.40–1.11)	0.123
Dairy						
Unqualified (<6)	Ref		Ref		Ref	
Qualified (≥6)	2.25 (1.47–3.47)	**<0.001**	1.95 (1.30–2.93)	**0.001**	2.12 (1.36–3.29)	**<0.001**
Fatty acids						
Unqualified (<6)	Ref		Ref		Ref	
Qualified (≥6)	0.94 (0.54–1.64)	0.832	0.84 (0.52–1.35)	0.477	0.95 (0.56–1.60)	0.843
Moderation components						
Refined grains						
Unqualified (<6)	Ref		Ref		Ref	
Qualified (≥6)	0.91 (0.55–1.53)	0.732	0.94 (0.61–1.44)	0.768	1.04 (0.61–1.77)	0.890
Sodium						
Unqualified (<6)	Ref		Ref		Ref	
Qualified (≥6)	1.32 (0.76–2.30)	0.322	1.28 (0.78–2.11)	0.330	1.53 (0.88–2.64)	0.129
Added sugars						
Unqualified (<6)	Ref		Ref		Ref	
Qualified (≥6)	0.70 (0.41–1.19)	0.186	0.53 (0.34–0.84)	**0.006**	0.44 (0.25–0.77)	**0.004**
Saturated fats						
Unqualified (<6)	Ref		Ref		Ref	
Qualified (≥6)	0.83 (0.51–1.36)	0.459	0.73 (0.47–1.14)	0.169	0.79 (0.52–1.22)	0.295

HEI-2020, Health Eating Index-2020; HR, hazard ratio; CI, confidence interval. Model 1: unadjusted model; Model 2: adjusted for age, and race; Model 3: age, race, marital status, educational level, family poverty income ratio, smoking status, alcohol use, BMI, hypertension, hyperlipidemia, diabetes, moderate or vigorous physical activity, and time between diagnosis and study entry. The bold *p*-values are less than 0.05, indicating statistical significance.

Notable associations were identified for certain HEI components. Participants with lower consumption of added sugars (with a qualified component score) demonstrated a reduced risk of all-cause mortality (HR 0.44, 95%CI: 0.25–0.77), whereas those with higher intake of dairy (with a qualified component score) faced an increased risk of all-cause mortality (HR 2.12, 95%CI: 1.36–3.29).

### 3.3 Association between HEI-2020 and cancer-specific mortality

In the fully adjusted model (model 3, Table 3), no significant differences were observed in the total HEI-2020 score or component scores between groups, except for the component “seafood and plant proteins.” Participants with higher consumption of seafood and plant proteins (with a qualified component score) exhibited a significantly increased risk of cancer-specific mortality (HR 3.64, 95% CI: 1.57–8.45).

**Table 3:** Association between HEI-2020 and cancer-specific mortality among breast cancer survivors (*n* = 481).

	Model 1		Model 2		Model 3	
	HR (95%CI)	*p-*value	HR (95%CI)	*p-*value	HR (95%CI)	*p-*value
Total HEI-2020 score						
Unqualified (<60)	Ref		Ref		Ref	
Qualified (≥60)	1.37 (0.73–2.59)	0.327	1.21 (0.69–2.12)	0.503	1.49 (0.84–2.66)	0.176
Adequacy components						
Total fruits						
Unqualified (<3)	Ref		Ref		Ref	
Qualified (≥3)	1.46 (0.78–2.71)	0.234	1.12 (0.63–2.00)	0.695	1.27 (0.70–2.30)	0.441
Whole fruits						
Unqualified (<3)	Ref		Ref		Ref	
Qualified (≥3)	0.78 (0.40–1.54)	0.480	0.65 (0.34–1.24)	0.188	0.78 (0.39–1.55)	0.482
Total vegetables						
Unqualified (<3)	Ref		Ref		Ref	
Qualified (≥3)	1.03 (0.42–2.56)	0.947	1.01 (0.42–2.44)	0.986	1.20 (0.46–3.14)	0.710
Greens and beans						
Unqualified (<3)	Ref		Ref		Ref	
Qualified (≥3)	0.97 (0.34–2.80)	0.962	1.02 (0.38–2.74)	0.975	1.17 (0.39–3.52)	0.779
Total protein foods						
Unqualified (<3)	Ref		Ref		Ref	
Qualified (≥3)	1.94 (0.41–9.17)	0.402	1.60 (0.41–6.22)	0.496	2.14 (0.62–7.36)	0.228
Seafood and plant proteins						
Unqualified (<3)	Ref		Ref		Ref	
Qualified (≥3)	2.01 (0.82–4.90)	0.126	2.22 (0.99–5.01)	0.054	3.64 (1.57–8.45)	**0.003**
Whole grains						
Unqualified (<6)	Ref		Ref		Ref	
Qualified (≥6)	0.88 (0.41–1.91)	0.749	0.75 (0.35–1.59)	0.448	0.67 (0.27–1.67)	0.395
Dairy						
Unqualified (<6)	Ref		Ref		Ref	
Qualified (≥6)	1.42 (0.75–2.67)	0.280	1.29 (0.68–2.45)	0.428	1.34 (0.69–2.62)	0.385
Fatty acids						
Unqualified (<6)	Ref		Ref		Ref	
Qualified (≥6)	1.40 (0.71–2.75)	0.332	1.30 (0.71–2.40)	0.393	1.75 (0.92–3.35)	0.090
Moderation components						
Refined grains						
Unqualified (<6)	Ref		Ref		Ref	
Qualified (≥6)	0.78 (0.36–1.69)	0.524	0.80 (0.37–1.71)	0.560	0.99 (0.50–1.96)	0.970
Sodium						
Unqualified (<6)	Ref		Ref		Ref	
Qualified (≥6)	1.05 (0.44–2.47)	0.918	0.97 (0.42–2.24)	0.938	1.11 (0.47–2.62)	0.805
Added sugars						
Unqualified (<6)	Ref		Ref		Ref	
Qualified (≥6)	1.25 (0.46–3.41)	0.661	1.09 (0.37–3.23)	0.881	1.16 (0.38–3.50)	0.795
Saturated fats						
Unqualified (<6)	Ref		Ref		Ref	
Qualified (≥6)	1.18 (0.60–2.31)	0.625	1.11 (0.57–2.13)	0.763	1.43 (0.68–2.99)	0.343

HEI-2020, Health Eating Index-2020; HR, hazard ratio; CI, confidence interval. Model 1: unadjusted model; Model 2: adjusted for age, and race; Model 3: age, race, marital status, educational level, family poverty income ratio, smoking status, alcohol use, BMI, hypertension, hyperlipidemia, diabetes, moderate or vigorous physical activity, and time between diagnosis and study entry. The bold *p*-values are less than 0.05, indicating statistical significance.

### 3.4 Association between HEI-2020 and non-cancer mortality

As shown in Table 4, model 3, a qualified total HEI-2020 score was significantly correlated with a reduction in non-cancer mortality (HR 0.59, 95% CI: 0.35–0.99) after full adjustments. Regarding HEI components, participants with lower intake of added sugars (with a qualified component score) experienced a decrease in non-cancer mortality (HR 0.25, 95% CI: 0.13–0.48). Conversely, participants with higher dairy intake (with a qualified component score) faced a marked increase in non-cancer mortality (HR 2.81, 95% CI: 1.56–5.07).

**Table 4:** Association between HEI-2020 and non-cancer mortality among breast cancer survivors (*n* = 481).

	Model 1		Model 2		Model 3	
	HR (95%CI)	*p-*value	HR (95%CI)	*p-*value	HR (95%CI)	*p-*value
Total HEI-2020 score						
Unqualified (<60)	Ref		Ref		Ref	
Qualified (≥60)	0.79 (0.47–1.34)	0.386	0.64 (0.40–1.04)	0.070	0.59 (0.35–0.99)	**0.047**
Adequacy components						
Total fruits						
Unqualified (<3)	Ref		Ref		Ref	
Qualified (≥3)	1.56 (0.95–2.55)	0.078	0.86 (0.51–1.43)	0.558	0.84 (0.49–1.45)	0.535
Whole fruits						
Unqualified (<3)	Ref		Ref		Ref	
Qualified (≥3)	1.35 (0.80–2.27)	0.267	0.94 (0.58–1.53)	0.801	0.92 (0.52–1.62)	0.762
Total vegetables						
Unqualified (<3)	Ref		Ref		Ref	
Qualified (≥3)	0.74 (0.37–1.47)	0.388	0.66 (0.36–1.22)	0.186	0.55 (0.28–1.08)	0.081
Greens and beans						
Unqualified (<3)	Ref		Ref		Ref	
Qualified (≥3)	0.60 (0.25–1.47)	0.264	0.70 (0.30–1.66)	0.421	0.74 (0.27–2.06)	0.569
Total protein foods						
Unqualified (<3)	Ref		Ref		Ref	
Qualified (≥3)	0.51 (0.22–1.16)	0.109	0.48 (0.22–1.07)	0.072	0.55 (0.22–1.35)	0.193
Seafood and plant proteins						
Unqualified (<3)	Ref		Ref		Ref	
Qualified (≥3)	0.64 (0.35–1.18)	0.152	0.74 (0.44–1.25)	0.263	0.74 (0.43–1.30)	0.299
Whole grains						
Unqualified (<6)	Ref		Ref		Ref	
Qualified (≥6)	1.03 (0.53–1.99)	0.931	0.75 (0.42–1.36)	0.341	0.68 (0.37–1.24)	0.207
Dairy						
Unqualified (<6)	Ref		Ref		Ref	
Qualified (≥6)	2.94 (1.70–5.09)	**<0.001**	2.50 (1.47–4.25)	**<0.001**	2.81 (1.56–5.07)	**<0.001**
Fatty acids						
Unqualified (<6)	Ref		Ref		Ref	
Qualified (≥6)	0.75 (0.39–1.45)	0.392	0.66 (0.37–1.18)	0.158	0.66 (0.33–1.31)	0.237
Moderation components						
Refined grains						
Unqualified (<6)	Ref		Ref		Ref	
Qualified (≥6)	1.00 (0.54–1.84)	0.996	1.05 (0.63–1.74)	0.855	1.11 (0.53–2.34)	0.778
Sodium						
Unqualified (<6)	Ref		Ref		Ref	
Qualified (≥6)	1.48 (0.77–2.86)	0.240	1.52 (0.83–2.82)	0.178	1.85 (0.90–3.80)	0.092
Added sugars						
Unqualified (<6)	Ref		Ref		Ref	
Qualified (≥6)	0.53 (0.28–1.01)	0.053	0.37 (0.20–0.66)	**<0.001**	0.25 (0.13–0.48)	**<0.001**
Saturated fats						
Unqualified (<6)	Ref		Ref		Ref	
Qualified (≥6)	0.69 (0.38–1.23)	0.206	0.57 (0.33–1.00)	**0.049**	0.54 (0.29–1.01)	0.055

HEI-2020, Health Eating Index-2020; HR, hazard ratio; CI, confidence interval. Model 1: unadjusted model; Model 2: adjusted for age, and race; Model 3: age, race, marital status, educational level, family poverty income ratio, smoking status, alcohol use, BMI, hypertension, hyperlipidemia, diabetes, moderate or vigorous physical activity, and time between diagnosis and study entry. The bold *p*-values are less than 0.05, indicating statistical significance.

### 3.5 Sensitivity analyses

Sensitivity analyses were performed in participants with complete data only (*N* = 419). Results also showed that qualified total HEI-2020 scores were significantly associated with reduced non-cancer mortality, but not with all-cause or cancer-specific mortality. Additionally, paradoxical findings in dairy, as well as seafood and plant proteins were also observed (Supplementary Table S1).

## 4 Discussion

The present study aimed to investigate the relationship between DQ as assessed by the HEI-2020 and mortality outcomes among BC survivors using data from the NHANES between 2005 and 2018. Our findings suggest that while a higher overall HEI-2020 score was significantly associated with reduced non-cancer mortality, it did not significantly correlate with all-cause or cancer-specific mortality. Additionally, paradoxical correlations were found in this population between an increased mortality risk and appropriate consumption of dairy, as well as seafood and plant proteins. Sensitivity analyses further confirmed the robustness of these findings.

The mixed results from our study differ from some previous cohort studies, such as that by George et al. (in 670 BC survivors) and Deshmukh et al. (in 131 BC survivors), who found significant associations between higher total HEI scores and reduced all-cause and cancer-specific mortality (15, 30). In contrast, our results were supported by Wang et al. (31) and Sun et al. (18), who found no significant association between total HEI scores and all-cause or cancer-specific mortality in a cohort of 3,450 BC survivors from China and another cohort of 2,295 postmenopausal female BC survivors from the Women’s Health Initiative in the USA. The variations in outcomes across studies may be partly attributed to differences in study populations and dietary assessment methodologies. More importantly, when further analyzing the relationship between HEI components and mortality outcomes, we found that appropriate consumption of certain foods under the guidance of HEI-2020 was paradoxically associated with a higher risk of mortality. For example, BC survivors with adequate intakes of seafood and plant proteins were instead at higher risk of cancer-specific mortality (HR 3.64), and survivors with adequate intakes of dairy products were instead at higher risk of all-cause and non-cancer mortality (HR 2.12 and 2.81, respectively). Thus, although the appropriate intake of some other foods was significantly associated with a lower mortality risk (e.g., limited intake of added sugars), the inclusion of all 13 HEI components (including dairy, as well as seafood and plant proteins) would likely diminish the benefit of limiting the intake of added sugars for BC survivors. Ultimately, no significant associations were observed between total HEI scores and all-cause or cancer-specific mortality in this study, suggesting the HEI-2020 may not fully capture the dietary needs specific to BC survivors.

The significant associations found in this study between limited added sugar intake and reduced all-cause as well as non-cancer mortality are consistent with previous studies (32–34). Farvid et al. prospectively assessed post-diagnostic intake of total sugar, added sugar, and natural sugar in a cohort of 8,932 BC survivors from the United States, and found that higher added sugar intake was associated with increased all-cause mortality (HR 1.20) over 11.5 years of follow-up (33). Another cohort study conducted in 927 BC survivors from the United States reported increased all-cause and BC-specific mortality (HR 1.62 and 1.85, respectively) in participants with higher consumption of sugar-sweetened soda drinks over 18.7 years of follow-up (34). Excessive consumption of added sugars introduces elevated insulin levels, thus promoting the growth and proliferation of BC cells, and indirectly regulating a range of factors, including insulin-like growth factors, sex hormones, and adipokines (32).

The relationship between post-diagnosis dairy consumption and mortality in BC survivors remains inconclusive, with a limited number of relevant studies available. The present study revealed that BC survivors who consumed sufficient dairy products exhibited an increased risk of all-cause and non-cancer mortality. This finding is not consistent with the DGA, but it was supported by a cohort study conducted by Kroenke et al. among 1,893 BC survivors, reporting that overall dairy intake was positively associated with all-cause mortality, and particularly high-fat dairy intake was positively correlated with both all-cause and non-cancer mortality (HR 1.20 and 1.49), over a median follow-up of 11.8 years (35). Moreover, numerous studies have explored the link between dairy consumption and BC incidence risk, yet the results are not entirely consistent (36, 37). While many investigations have suggested a negative association between dairy intake and BC risk (38, 39), Kaluza et al. conducted a more nuanced analysis by categorizing dairy products into fermented and non-fermented types and reported different results in a population-based Swedish Mammography Cohort including 33,780 women (40). Their findings indicated that high long-term consumption of milk was linked to an elevated risk of estrogen receptor-positive (ER+)/progesterone receptor-positive (PR+) BC in postmenopausal women, whereas high long-term consumption of fermented dairy products was associated with a reduced risk of estrogen receptor-negative (ER-)/progesterone receptor-negative (PR-) BC. Another two-arm population-based case–control study involving 823 BC cases and 876 controls in Polish women found individual dairy products have a statistically significant but bi-directional relationship with BC risk, which differs between premenopausal and postmenopausal women (41). In summary, the relationship between dairy products and BC remains inconclusive, necessitating further investigation into the effects of types of dairy products, BC subtypes, menopausal status, and other influencing factors.

The HEI-2020 component “seafood and plant proteins” primarily encompasses seafood, soy and soy products, as well as nuts (13). The present study indicates that BC survivors who consume a sufficient quantity of “seafood and plant proteins” experienced an elevated risk of cancer-specific mortality. For now, we are unable to determine which food or combination of foods from the component brought BC survivor a higher cancer-specific mortality risk. Prior research has indicated that the post-diagnosis intake of soy products could enhance the prognosis for survivors, potentially decreasing the risk of mortality and recurrence (42, 43). However, some other studies failed to demonstrate statistically significant correlations between soy consumption and mortality, including a study based on 9,514 BC survivors from two US cohorts and one Chinese cohort during 1991 and 2006 (44). Furthermore, meta-analyses have suggested the protective effects of soy foods against BC may be predominantly applicable to Asian women (22, 45). In terms of fish consumption, laboratory evidence suggests that *ω*-3 polyunsaturated fatty acids (PUFAs) reduce inflammatory eicosanoids produced by the metabolism of ω-6 PUFA through competitive inhibition, and that the ω-3-induced cytotoxic milieu increases apoptosis and reduces cell growth in BC cells (23, 46, 47). However, epidemiologic investigations on whether dietary ω-3 PUFA intake, of which fish is a major source, is beneficial for BC survivors are limited and inconsistent (48, 49). Moreover, although a number of studies found a negative relationship between fish consumption and BC incidence (50, 51), there are some studies conversely reporting a positive association between fish intake and BC incidence, including a prospective cohort study in 23,693 postmenopausal women, and another population-based case–control study reporting significant association between tuna intake and BC risk (OR 1.25, 95% CI 1.05–1.50) in US Non-Hispanic white women (52, 53). The link between seafood (including fish) consumption and higher BC risk may be partly related to microplastic contamination. Studies suggest that microplastic contamination can cause toxicity, oxidative stress, inflammation, and increased particle uptake (54, 55). The immune system’s inability to remove synthetic particles may lead to chronic inflammation and a higher risk of neoplasia. Additionally, the impact of these foods on BC survivors may also be modulated by factors such as cooking methods, food preservation techniques, cancer stage, and cancer subtype. In conclusion, epidemiological evidence regarding the relationship between seafood, soy and soy products, nuts, with BC mortality remains inconclusive. Consequently, no conclusions can be drawn as to which food within this component may pose an increased mortality risk for BC survivors, nor to determine whether this component is associated with mortality outcomes in this population. Notably, to our knowledge, there is a lack of studies that have specifically examined the level of this HEI-2020 component (seafood and plant proteins) and its correlation with mortality outcomes in BC survivors. Further epidemiological and mechanistic investigations are warranted to address these gaps in knowledge.

The unexpected associations observed in our study suggest that certain components of a diet considered healthy in the general population may not be as beneficial for BC survivors, and the HEI-2020 might not be an appropriate indicator for post-diagnosis dietary assessment or recommendations in this group.

One of the strengths of this study is the use of NHANES data, which is nationally representative and reflects the diversity of the U.S. population. In addition, a series of covariates were controlled in the study, including demographic characteristics, socioeconomic factors, comorbidities, and lifestyle factors, to help reduce potential confounding bias. However, it is also important to acknowledge the limitations inherent in the present study to appropriately contextualize the findings. Firstly, since the data regarding diet and cancer diagnosis were obtained from self-reported questionnaires, recall bias may exist to some extent. Secondly, although the study sample is nationally representative, missing data on diet and covariates may still introduce bias to some degree. Thirdly, due to limitations of the study dataset, the analysis did not include confounders such as cancer stage, cancer subtype, and treatment modalities, which may limit the precision of the inferences drawn from the results. Lastly, this study assessed the HEI-2020 of BC survivors only at baseline, without conducting subsequent assessments throughout the follow-up period, which may restrict the understanding of long-term dietary patterns and their effects on health outcomes. Future studies should conduct repeated dietary assessments and include additional confounders to yield more comprehensive results.

## 5 Conclusion

The current study suggests that although higher HEI-2020 scores may correlate with certain mortality benefits in BC survivors, the relationships are not uniformly favorable. These findings challenge the applicability of the HEI-2020 for post-diagnosis DQ assessment for BC survivors, and underscore the need for additional research into the specific dietary requirements and health outcomes in this demographic, as well as the underlying mechanism involved, to develop more targeted dietary recommendations and assessment indicators for this population.

## Data Availability

Publicly available datasets were analyzed in this study. This data can be found here: NHANES Questionnaires, Datasets, and Related Documentation. https://wwwn.cdc.gov/nchs/nhanes/default.aspx (accessed 2024-10-21).
