# Supplementary material for: The healthy eating index may not be an appropriate indicator for assessing dietary quality in breast cancer survivors: results from NHANES 2005–2018
Source: Front Nutr. 2024 Dec 30;11:1519607. doi: 10.3389/fnut.2024.1519607 (PMC11725467; doi:10.3389/fnut.2024.1519607)
Supplement: Supplementary file 1 [file Data_Sheet_1.pdf]

Supplementary Material

Supplementary Table

Table S1. Association between HEI-2020 and mortality among breast cancer survivors.

	All-cause mortality		Cancer mortality		Noncancer mortality	
	HR (95%CI)	*P*-value	HR (95%CI)	*P*-value	HR (95%CI)	*P*-value
**Total HEI-2020 score**						
Unqualified (<60)	Ref		Ref		Ref	
Qualified (≥60)	0.74 (0.46-1.20)	0.221	1.38 (0.69-2.76)	0.360	0.51 (0.28-0.94)	**0.031**
**Adequacy components**						
Total fruits						
Unqualified (<3)	Ref		Ref		Ref	
Qualified (≥3)	1.05 (0.63-1.77)	0.843	1.67 (0.81-3.41)	0.162	0.75 (0.35-1.62)	0.467
Whole fruits						
Unqualified (<3)	Ref		Ref		Ref	
Qualified (≥3)	1.05 (0.65-1.70)	0.850	1.00 (0.44-2.27)	0.993	1.02 (0.50-2.09)	0.950
Total vegetables						
Unqualified (<3)	Ref		Ref		Ref	
Qualified (≥3)	0.78 (0.45-1.35)	0.374	1.16 (0.36-3.75)	0.808	0.62 (0.32-1.21)	0.163
Greens and beans						
Unqualified (<3)	Ref		Ref		Ref	
Qualified (≥3)	0.80 (0.34-1.90)	0.616	1.11 (0.33-3.75)	0.864	0.66 (0.19-2.36)	0.523
Total protein foods						
Unqualified (<3)	Ref		Ref		Ref	
Qualified (≥3)	0.84 (0.38-1.86)	0.667	2.03 (0.64-6.38)	0.227	0.60 (0.22-1.59)	0.302
Seafood and plant proteins						
Unqualified (<3)	Ref		Ref		Ref	
Qualified (≥3)	1.68 (1.02-2.77)	**0.042**	4.07 (1.73-9.59)	**0.001**	0.89 (0.47-1.68)	0.712
Whole grains						
Unqualified (<6)	Ref		Ref		Ref	
Qualified (≥6)	0.80 (0.48-1.33)	0.390	0.59 (0.21-1.68)	0.321	0.95 (0.55-1.65)	0.863
Dairy						
Unqualified (<6)	Ref		Ref		Ref	
Qualified (≥6)	1.94 (1.18-3.20)	**0.009**	1.56 (0.70-3.47)	0.278	2.32 (1.23-4.38)	**0.009**
Fatty acids						
Unqualified (<6)	Ref		Ref		Ref	
Qualified (≥6)	0.90 (0.50-1.60)	0.708	1.80 (0.73-4.41)	0.202	0.54 (0.26-1.14)	0.105
**Moderation components**						
Refined grains						
Unqualified (<6)	Ref		Ref		Ref	
Qualified (≥6)	1.00 (0.55-1.83)	0.999	1.03 (0.48-2.24)	0.933	1.06 (0.47-2.36)	0.894
Sodium						
Unqualified (<6)	Ref		Ref		Ref	

Qualified (≥6)	1.67 (0.89-3.12)	0.108	1.13 (0.39-3.24)	0.817	2.25 (1.10-4.63)	**0.027**
Added sugars						
Unqualified (<6)	Ref		Ref		Ref	
Qualified (≥6)	0.44 (0.24-0.82)	**0.010**	0.84 (0.35-2.01)	0.703	0.28 (0.13-0.62)	**0.002**
Saturated fats						
Unqualified (<6)	Ref		Ref		Ref	
Qualified (≥6)	0.80 (0.53-1.21)	0.287	1.37 (0.64-2.96)	0.418	0.57 (0.33-0.98)	**0.041**

HEI-2020, Health Eating Index-2020; HR, hazard ratio; CI, confidence interval.
Models were adjusted for age, race, marital status, educational level, family poverty income ratio, smoking status, alcohol use, BMI, hypertension, hyperlipidemia, diabetes, moderate or vigorous physical activity, and time between diagnosis and study entry.
The bold *P*-values are less than 0.05, indicating statistical significance.
