# Supplementary material for: The healthy eating index may not be an appropriate indicator for assessing dietary quality in breast cancer survivors: results from NHANES 2005–2018
Source: Front Nutr. 2024 Dec 30;11:1519607. doi: 10.3389/fnut.2024.1519607 (PMC11725467; doi:10.3389/fnut.2024.1519607)
Supplement: Supplementary file 2 [file Table_1.docx]

Supplementary Material

# Supplementary Table

Table S1. Association between HEI-2020 and mortality among breast cancer survivors.

|  | | All-cause mortality | |  | Cancer mortality | |  | Noncancer mortality | |
| --- | --- | --- | --- | --- | --- | --- | --- | --- | --- |
|  |  | HR (95%CI) | *P-*value |  | HR (95%CI) | *P-*value |  | HR (95%CI) | *P-*value |
| **Total HEI-2020 score** | |  |  |  |  |  |  |  |  |
|  | Unqualified (<60) | Ref |  |  | Ref |  |  | Ref |  |
|  | Qualified (≥60) | 0.74 (0.46-1.20) | 0.221 |  | 1.38 (0.69-2.76) | 0.360 |  | 0.51 (0.28-0.94) | **0.031** |
| **Adequacy components** | |  |  |  |  |  |  |  |  |
| Total fruits | |  |  |  |  |  |  |  |  |
|  | Unqualified (<3) | Ref |  |  | Ref |  |  | Ref |  |
|  | Qualified (≥3) | 1.05 (0.63-1.77) | 0.843 |  | 1.67 (0.81-3.41) | 0.162 |  | 0.75 (0.35-1.62) | 0.467 |
| Whole fruits | |  |  |  |  |  |  |  |  |
|  | Unqualified (<3) | Ref |  |  | Ref |  |  | Ref |  |
|  | Qualified (≥3) | 1.05 (0.65-1.70) | 0.850 |  | 1.00 (0.44-2.27) | 0.993 |  | 1.02 (0.50-2.09) | 0.950 |
| Total vegetables | |  |  |  |  |  |  |  |  |
|  | Unqualified (<3) | Ref |  |  | Ref |  |  | Ref |  |
|  | Qualified (≥3) | 0.78 (0.45-1.35) | 0.374 |  | 1.16 (0.36-3.75) | 0.808 |  | 0.62 (0.32-1.21) | 0.163 |
| Greens and beans | |  |  |  |  |  |  |  |  |
|  | Unqualified (<3) | Ref |  |  | Ref |  |  | Ref |  |
|  | Qualified (≥3) | 0.80 (0.34-1.90) | 0.616 |  | 1.11 (0.33-3.75) | 0.864 |  | 0.66 (0.19-2.36) | 0.523 |
| Total protein foods | |  |  |  |  |  |  |  |  |
|  | Unqualified (<3) | Ref |  |  | Ref |  |  | Ref |  |
|  | Qualified (≥3) | 0.84 (0.38-1.86) | 0.667 |  | 2.03 (0.64-6.38) | 0.227 |  | 0.60 (0.22-1.59) | 0.302 |
| Seafood and plant proteins | | |  |  |  |  |  |  |  |
|  | Unqualified (<3) | Ref |  |  | Ref |  |  | Ref |  |
|  | Qualified (≥3) | 1.68 (1.02-2.77) | **0.042** |  | 4.07 (1.73-9.59) | **0.001** |  | 0.89 (0.47-1.68) | 0.712 |
| Whole grains | |  |  |  |  |  |  |  |  |
|  | Unqualified (<6) | Ref |  |  | Ref |  |  | Ref |  |
|  | Qualified (≥6) | 0.80 (0.48-1.33) | 0.390 |  | 0.59 (0.21-1.68) | 0.321 |  | 0.95 (0.55-1.65) | 0.863 |
| Dairy | |  |  |  |  |  |  |  |  |
|  | Unqualified (<6) | Ref |  |  | Ref |  |  | Ref |  |
|  | Qualified (≥6) | 1.94 (1.18-3.20) | **0.009** |  | 1.56 (0.70-3.47) | 0.278 |  | 2.32 (1.23-4.38) | **0.009** |
| Fatty acids | |  |  |  |  |  |  |  |  |
|  | Unqualified (<6) | Ref |  |  | Ref |  |  | Ref |  |
|  | Qualified (≥6) | 0.90 (0.50-1.60) | 0.708 |  | 1.80 (0.73-4.41) | 0.202 |  | 0.54 (0.26-1.14) | 0.105 |
| **Moderation components** | | |  |  |  |  |  |  |  |
| Refined grains | |  |  |  |  |  |  |  |  |
|  | Unqualified (<6) | Ref |  |  | Ref |  |  | Ref |  |
|  | Qualified (≥6) | 1.00 (0.55-1.83) | 0.999 |  | 1.03 (0.48-2.24) | 0.933 |  | 1.06 (0.47-2.36) | 0.894 |
| Sodium | |  |  |  |  |  |  |  |  |
|  | Unqualified (<6) | Ref |  |  | Ref |  |  | Ref |  |
|  | Qualified (≥6) | 1.67 (0.89-3.12) | 0.108 |  | 1.13 (0.39-3.24) | 0.817 |  | 2.25 (1.10-4.63) | **0.027** |
| Added sugars | |  |  |  |  |  |  |  |  |
|  | Unqualified (<6) | Ref |  |  | Ref |  |  | Ref |  |
|  | Qualified (≥6) | 0.44 (0.24-0.82) | **0.010** |  | 0.84 (0.35-2.01) | 0.703 |  | 0.28 (0.13-0.62) | **0.002** |
| Saturated fats | |  |  |  |  |  |  |  |  |
|  | Unqualified (<6) | Ref |  |  | Ref |  |  | Ref |  |
|  | Qualified (≥6) | 0.80 (0.53-1.21) | 0.287 |  | 1.37 (0.64-2.96) | 0.418 |  | 0.57 (0.33-0.98) | **0.041** |
| HEI-2020, Health Eating Index-2020; HR, hazard ratio; CI, confidence interval.  Models were adjusted for age, race, marital status, educational level, family poverty income ratio, smoking status, alcohol use, BMI, hypertension, hyperlipidemia, diabetes, moderate or vigorous physical activity, and time between diagnosis and study entry. The bold *P*-values are less than 0.05, indicating statistical significance. | | | | | | | | | |
